# The Role of Apical Periodontitis Disease in the Development of Bisphosphonate-Related Osteonecrosis of the Jaw: An Animal Study

**DOI:** 10.3390/dj11070168

**Published:** 2023-07-12

**Authors:** Manuel Marques-Ferreira, Ana Margarida Abrantes, Anabela Paula, Mafalda Laranjo, Ana Salomé Pires, Francisco Caramelo, Juan José Segura-Egea, Ana Brito, Lina Carvalho, Maria Filomena Botelho, Eunice Carrilho, Carlos Miguel Marto, Siri Paulo

**Affiliations:** 1 Univ. Coimbra, Coimbra Institute for Clinical and Biomedical Research (iCBR), Area of Environment, Genetics and Oncobiology (CIMAGO), Faculty of Medicine, 3000-548 Coimbra, Portugal; mmferreira@fmed.uc.pt (M.M.-F.); mabrantes@fmed.uc.pt (A.M.A.); anabelabppaula@sapo.pt (A.P.); mafaldalaranjo@fmed.uc.pt (M.L.); pireslourenco@uc.pt (A.S.P.); fcaramelo@fmed.uc.pt (F.C.); abrito@ipn.pt (A.B.); lcarvalho@huc.min-saude.pt (L.C.); mfbotelho@fmed.uc.pt (M.F.B.); ecarrilho@fmed.uc.pt (E.C.); cmiguel.marto@uc.pt (C.M.M.); 2 Univ. Coimbra, Institute of Endodontics, Faculty of Medicine, 3000-548 Coimbra, Portugal; 3 Univ. Coimbra, Center for Innovative Biomedicine and Biotechnology (CIBB), 3004-504 Coimbra, Portugal; 4 Clinical Academic Center of Coimbra (CACC), 3004-561 Coimbra, Portugal; 5 Univ. Coimbra, Institute of Biophysics, Faculty of Medicine, 3000-548 Coimbra, Portugal; 6 Univ. Coimbra, Institute of Integrated Clinical Practice and Laboratory for Evidence-Based Sciences and Precision Dentistry (LACBE–MDP), Faculty of Medicine, 3000-548 Coimbra, Portugal; 7 Department of Stomatology (Endodontics Section), University of Sevilla, 41009 Sevilla, Spain; segurajj@us.es; 8 Univ. of Coimbra, IAP, Faculty of Medicine, 3004-504 Coimbra, Portugal; 9 Univ. of Coimbra, Institute of Experimental Pathology, Faculty of Medicine, 3000-548 Coimbra, Portugal

**Keywords:** bone loss, medication-related osteonecrosis of the jaw, nuclear medicine, periapical lesions, zoledronate therapy

## Abstract

Background: Microorganisms and their by-products are responsible for establishing pulpal and periapical diseases. Healing is compromised in patients under bisphosphonate therapy, and the presence of periapical infections can potentially lead to the development of medication-related osteonecrosis of the jaw (MRONJ). This work aimed to evaluate if bisphosphonate therapy is a risk factor for MRONJ development in the presence of periapical lesions. Methods: Two groups of 10 female Wistar rats were used. The experimental group received zoledronate (0.1 mg/kg) intraperitoneally, and the control received a saline solution, three times a week for three weeks. One week after the last injection, apical periodontitis was induced through pulpal exposure in the mandibular first molars. Twenty-one days later, the animals were intravenously injected with ^99m^Tc-HMDP, and the radioactivity uptake by mandibular specimens was counted. In addition, sample radiographs and a histological examination were performed. Results: The bone loss was higher in the control group when compared to the experimental group (*p* = 0.027). ^99m^Tc-HMDP uptake in the control was reduced compared with the experimental group, although without statistical significance. Conclusions: In the presence of zoledronate therapy, apical periodontitis does not increase the risk of MRONJ development, and periapical lesions have lower bone resorption when compared to the control group.

## 1. Introduction

Dental-pulp exposure to microorganisms can happen through an open-pulp chamber by caries, infiltration of previous restorations, or periodontal lesions that communicate with the pulp space. Consequently, root-canal-system colonization by microorganisms occurs. Additionally, the substances released by microorganisms, such as toxins, combined with pro-inflammatory cytokines, lead to pulp inflammation and eventual necrosis, followed by a periapical inflammatory response [1,2]. 

Several microorganism types were found in endodontic infections, such as fungi, archaea, and viruses; however, bacteria are predominant [3]. The Gram-positive *Actinomyces* sp. are one of the most common and resistant species because of their ability to aggregate and resist phagocytosis. They are present in the oral cavity flora and can colonize the pulp space and contribute to periapical lesion development [4]. Other Gram-positive anaerobes usually present are *Enterococcus faecalis* sp., *Propionibacterium* sp., and *Streptococcus* sp., frequently associated with *Actinomyces* sp. and *Staphylococcus* sp. when fistulas are present. The Gram-negative anaerobe *Fusobacterium nucleatum* sp., and *Prevotella* sp., as well as *Porphyromona gingivalis* sp., are responsible for purulent abscesses [5,6,7]. When periapical lesions are present, other species can also be detected. In a rat animal model, *Enterococcus faecalis* sp. and *Fusobacterium nucleatum* sp. were found in induced apical periodontitis and, in chronic apical periodontitis, *Porphyromonas gingivalis* sp. was also detected [8]. 

If the pulp infection remains untreated, a periapical lesion will occur as a response to the bacterial colonization and immune-system activation. This is due to the recruitment of inflammatory cells and activation of osteoclasts in a process regulated by osteoclast and osteoblast dynamics and transcription factors, which include the receptor activator of the NF-κB ligand (RANKL) and receptor activator of NF-κB (RANK) [9]. Consequently, periapical bone resorption occurs, which can be radiographically identified as a radiolucent periapical lesion [1,10,11].

Besides the local factors described, systemic diseases can also influence endodontic infections. It has been shown that the incidence of endodontic infection in compromised patients is higher when compared with a population without disorders [12,13]. 

Bisphosphonates (BPs) are pharmacological agents commonly used to treat bone-related conditions, such as osteoporosis, and to prevent and treat bone metastasis in solid tumours. The mechanism of action is based on the BPs’ high affinity with bone, leading to osteoclast apoptosis and bone-resorption inhibition [14,15,16,17]. Consequently, they decrease bone turnover and reduce bone metastases [14,15,16,17]. There are two classes of bisphosphonates, regarding the presence or absence of nitrogen in the molecular structure. Zoledronate (ZOL) is the most widely used among these drugs since it presents higher clinical efficacy [16,17]. However, since they interfere with the bone healing process, BPs have been linked to an increased risk of medication-related osteonecrosis of the jaw (MRONJ) [14,15,16,17]. MRONJ is an area of exposed necrotic bone and inflammation of the surrounding tissue that has been present for more than 8 weeks [18,19,20]. 

In recent years, the number of patients who need dental treatments and to whom bisphosphonates are prescribed has increased [14,15,16,17], and several reports have described MRONJ as a clinically adverse side effect in patients under BP therapy when invasive dental procedures, such as oral surgeries, are performed in teeth with previous active infection [18]. Also, although guidelines exist for managing such cases, they are mainly based on conservative treatment for pain control, infection reduction, and minimizing bone-necrosis progression [18,19,20]. Consequently, MRONJ is an increasing oral health problem, and the comprehension of its aetiology and prevention is particularly important. Regarding endodontics, there is a lack of knowledge about the influence of BPs in the development of apical periodontitis and in the outcomes of endodontic treatment, and also about the relationship between periapical infection and MRONJ [21]. However, an interrelationship between these factors can be accepted since local inflammation, bone resorption microenvironment, and compromised blood flow to the area in periapical lesions can contribute to osteonecrosis in susceptible patients undergoing BP therapies [18,19,20]. 

To clarify the role of apical periodontitis in MRONJ development, this study aimed to evaluate in vivo if experimentally induced endodontic periapical lesions contribute to the development of MRONJ after BP administration. The null hypothesis of this study is that the periapical lesion does not contribute to the establishment of MRONJ.

## 2. Materials and Methods

### 2.1. Animal Model

Twenty female Wistar rats, 17 to 20 weeks old and with an average weight of 210–230 g, were used in this study. The animals were housed in separate ventilated cages, ensuring proper air circulation in a controlled atmosphere at 20 ± 0.5 °C, 55 ± 10% humidity. They were exposed to a consistent light/dark cycle of 12 h each and had unrestricted access to a standard diet and filtered water-food and water ad libitum. To enhance their environment, paper rolls and strips were provided for their amusement and engagement. The animals were randomly allocated to different study groups and distinguished by earmarking. Regular welfare checks were conducted three times a week, and no significant problems were encountered during the entire duration of the study.

The animals were acquired from the Institute for Clinical and Biomedical Research (iCBR) animal facility. The experimental procedures were approved by the Animal Welfare Committee of the Faculty of Medicine (ref. 005-CE-2014 ), University of Coimbra, and were performed according to the international legislation and guidelines for animal research.

The rats were divided into control (Control Group—CT) and experimental (Zoledronate Group—ZOL) groups. After acclimatization, three intraperitoneal administrations per week were administrated over three weeks: zoledronate (ZOL) (0.1 mg/kg) to the ZOL group or saline solution (SS) to the CT group [22].

One week after the last injection of ZOL or SS, the rats were anesthetized with an intramuscular administration of xylazine and ketamine, 0.5 mL/kg body weight, and the pulps of the left mandibular first molars were exposed using a # ½ round bur to a depth equable to the diameter of the bur, to avoid furcal perforation [23]. The pulp was fragmented with a #15 K file, and teeth were exposed and left open to the oral environment during the experiment to develop apical periodontitis.

### 2.2. Nuclear Medicine Studies

After 21 days of pulp exposure, the animals were anesthetized as previously described and were injected intravenously with 40 ± 17 MBq of ^99m^Technetium hydroxy methylene diphosphonate (^99m^Tc-HMDP) for scintigraphy analysis. The bone scintigraphy acquisition was performed in a gamma camera GE Millennium three hours after administration. After this procedure, the animals were occised with an intravenous overdose of 100 mg/kg of body weight pentobarbital. The mandibles were carefully removed, and a static image was acquired for five minutes in the ZOL and CT groups, performed in a gamma camera GE Millennium, for each mandible. The regions of interest (ROIs) were drawn over each mandible to calculate uptake activity (counts per minute, cpm).

After nuclear medicine imaging, the jaws were inspected for ulcerated mucosa and bone exposure. Then, the jaws were cleaned of the soft tissues and placed in 4% paraformaldehyde for 48 h for tissue fixation. After this contact period, radiographs were taken with a parallel technique and performed with a dental X-ray unit (PORT –X II, Genoray, Seongnam-City, Gyeonggi-Do, Korean), with an exposure of 0.02 s (60 kVp, 2 mA) and the acquisition was made with a digital radiographic system (RVG Gendex, software VixWin Pro Version 1.5, KaVo Dental Gendex Dental Systems GmbH Germany 22761 Hamburg, Germany).

### 2.3. Radiographic Analysis

The radiographs were analysed using ImageJ 1.30 (Image Processing and Analysis in Java—National Institutes of Health, Bethesda, MD, USA), and their area was calculated using the delineation of the periapical lesions.

### 2.4. Histological Analysis

After being radiographed, the mandibles were decalcified in a 10% EDTA/phosphate-buffered saline solution for four weeks and embedded in paraffin. Frontal sections in a mesiodistal direction of 6 µm thick were obtained and stained with haematoxylin and eosin (HE). One of four sequential sections that included the root of the first left mandibular molars was observed under a light microscope (Nikon Eclipse 80i, Nikon Corporation, Tokyo, Japan) and scanned using NIS-Elements software (Nikon Instruments Europe BV Postbus 769211070 KE, Amsterdam, The Netherlands). An observer, blinded to the analysed group, outlined the periapical lesion for each sample. Each image’s periapical lesion size values were measured in pixels with the software ImageJ 1.30. A qualitative analysis of the periapical region was also performed, focused on inflammatory infiltrate, bone and root resorption, and periodontal ligament alterations.

### 2.5. Statistical Analysis

The sample size (*n* = 20) was calculated to compare the means in two independent samples. A two-sided significance level of 5% (α = 0.05, Zα = 1.960), and 80% power (β = 0.20, Zβ = 0.842) was considered to detect a statistically significant difference and a hypothesized difference between the means of 15 points of the two groups. The results of all measurements were presented as mean values ± standard deviation, the statistical differences between groups were subjected to a non-parametric Mann–Whitney test, and values of *p* < 0.05 were considered statistically significant.

## 3. Results

During this study period, the animals in the two groups increased in weight, no behaviour changes or signs of animal stress were detected, and all animals tolerated the operative procedures with no animal loss. Twenty-one days after pulp exposure, all the rats from Zoledronate group and Control group clinically showed the epithelium without signs of inflammation and the absence of bone exposure during a clinical examination.

### 3.1. Scintigraphic Analysis

**Figure 1:**
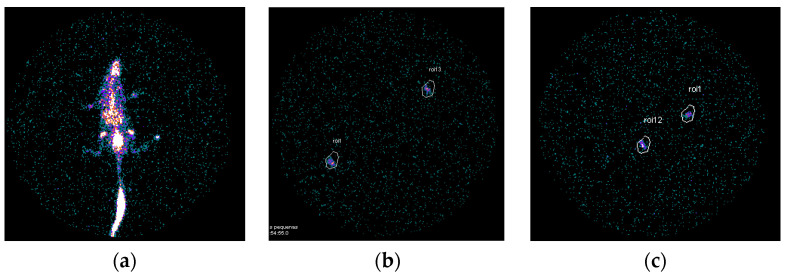
Scintigraphic representative images of the whole rat body (**a**) and mandible of ZOL group (**b**) and CT group (**c**) with ROIs and uptake of ^99m^Tc-HMDP.

**Figure 2:**
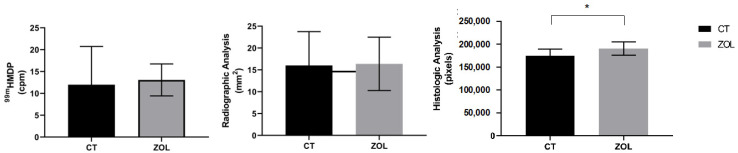
Radiographic, histologic, and nuclear medicine analysis. (Cpm = counts per minute; CT = control group (saline solution); ZOL = zoledronate). Statistical analysis: * *p* < 0.05.

After the experimental period, nuclear medicine images were performed (Figure 1a) and after occision, mandibles were taken, the regions of interest (ROIs) were drawn over each mandible (Figure 1b,c) and the counts per minute were acquired. The results showed an increased uptake of ^99m^Tc-HMDP of 13.08 ± 3.65 counts per minute (cpm) in the ZOL group. In contrast, in the CT group, the uptake was reduced to 11.98 ± 8.75 cpm, although with no statistical difference (*p* = 0.719) (Figure 2).

### 3.2. Radiographic Analysis

**Figure 3:**
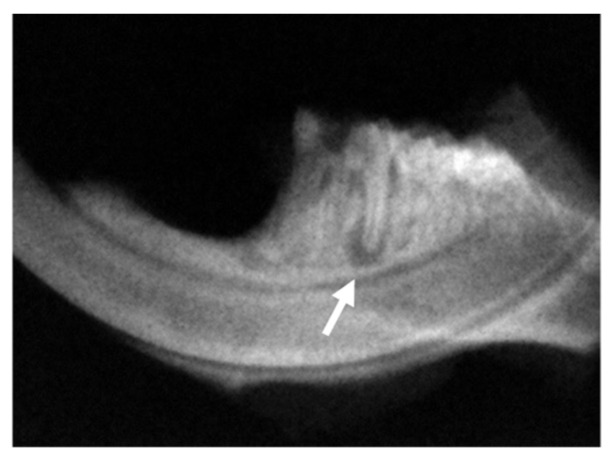
Radiographic images that show radiolucency developed at the apex (arrow) and furcation periodontium of the pulp-exposed tooth of the left mandible. The image is representative from an animal of the control group.

After the scintigraph assessment, the mouse mandibles were imaged after 21 days of pulpal exposure, as previously described. Radiolucency that appeared at the apex and furcation periodontium of the tooth with the exposed pulp was observed in the radiographic images (Figure 3). The radiographs were analysed using ImageJ 1.30 (Image Processing and Analysis in Java—National Institutes of Health, Bethesda, MD, USA) to quantify the amount of periapical bone loss. The periapical lesion was delimited in each image, and the area was determined in pixels.

The ZOL group demonstrated reduced bone resorption (16.01 ± 7.74 mm^2^) compared with the CT group 16.39 ± 6.10 mm^2^, but with no statistically significant difference (*p* = 0.903) (Figure 2).

### 3.3. Histologic and Histomorphometry Analysis

**Figure 4:**
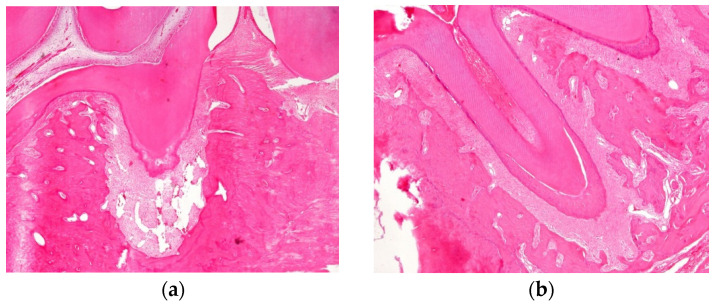
(**a**) Histological analysis exhibited an increased periodontal ligament with loss of lamina dura and alveolar bone resorption was considerably enlarged in the saline-solution group (190,515 ± 14,355 pixels). Magnification: 40×. (**b**) Histologic analysis exhibited an increased periodontal ligament with loss of lamina dura and alveolar bone resorption which was reduced in the ZOL group.

Twenty-one days after the periapical lesion inductions, an increased periodontal ligament with loss of lamina dura and alveolar bone resorption was observed. In addition, the lesion area had considerably enlarged in the saline solution group (190,515 ± 143,55 pixels), whereas in the ZOL group, it was reduced (174,822 ± 14,808 pixels), with statistical differences (*p* = 0.027) (Figure 2 and Figure 4).

**Figure 5:**
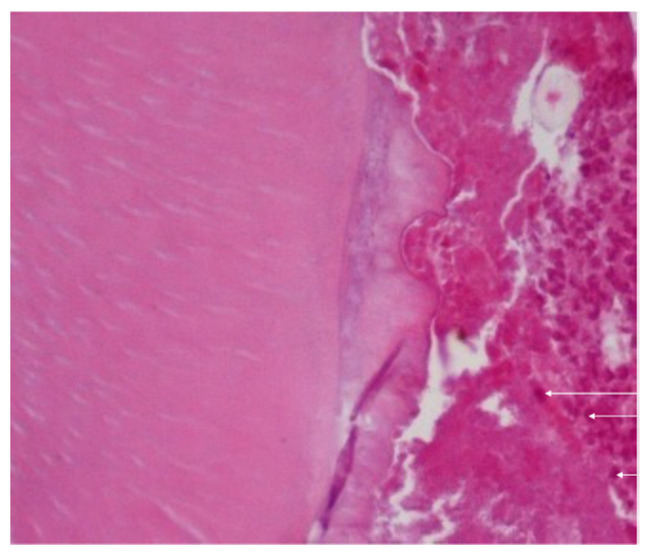
Histological analysis in all groups also exhibited periapical lesions with enlargement of the periodontal space and disorganization of the periodontal ligaments, with large number of macrophages present throughout the periodontal ligament (arrow), and no osteonecrosis was observed. Magnification: 400×.

At this time point, histological analysis in all groups also exhibited periapical lesions with enlargement of the periodontal space and disorganization of the periodontal ligaments, with large numbers of macrophages present throughout the periodontal ligament, and no osteonecrosis was observed (Figure 5). In addition, fibres and blood vessels occupied the lost bone space.

## 4. Discussion

This study investigated whether periapical infections contribute to osteonecrosis development after BP administration. Several studies have reported that zoledronate reduces bone resorption [24,25]; however, it is unclear whether periapical disease increases the risk of MRONJ. The experimental model chosen is a well-validated animal model, which correlates the administration of bisphosphonates for three weeks with the development of maxillary osteonecrosis after tooth extraction [22,26,27]. In the present animal model, adult female rats were used because female patients are more exposed to bisphosphonate therapy than male patients [28].

Periradicular bone destruction in all groups indicated that alveolar bone resorption was successfully induced due to the pulpal exposure followed by oral-flora contamination of the pulpal space and consequent inflamed periapical tissues, the onset of lesions and their extension into chronic lesions [10]. This is due to the mobilization of the host defense mechanism that aim’s to kill bacteria and prevent extraradicular invasion. However, this may not be limited to killing the microorganisms that invade the tooth but may also destroy tissue components and induce bone absorption [29].

Lin et al. [21] and Yoneda et al. [30] described that in a rat model, a rapid period of lesion expansion and bone destruction occurred between day 0 and day 14 after pulpal exposure, and the size of the periapical lesions can remain stable afterward [21,30]. In the first stage, lesions develop through bone resorption; they are filled with granulomatous tissue and with a barrier of polymorphonuclear leukocytes (PMN). Later, the periradicular lesions present as granulomas composed of solid soft tissue or as cysts with a semi-solid area [31]. In this study, a period of 21 days was chosen as the study period that guarantees the presence of a periapical lesion and the potential presence of an osteonecrosis lesion [30,32,33]. A decrease in bone loss in the ZOL group was histologically more evident than in the CT group, with significant differences (*p* = 0.027). This supports the theory that the BP effect diminished bone resorption in the experimental periapical lesions.

The antiresorptive effect of this drug can explain this result, so it can be hypothesized that ZOL may have a protective effect against bone loss in this experimental model. Several reports exist regarding the cytokines involved in bone metabolism regulation [21,34,35]. Among those, key molecules such as RANKL, RANK, and OPG, the new TNF ligand and receptor-signalling family members, are final effectors of osteoclast differentiation and function [34,36].

ZOL inhibits the differentiation of osteoclasts by suppressing the RANKL/RANK pathways and inhibiting macrophage differentiation into osteoclasts. Additionally, it induces osteoclast apoptosis by inhibiting the farnesyl pyrophosphate synthase-mediated mevalonate pathway [24,36]. The mechanism by which the zoledronate attenuates the bone resorption, seen in the histology, remains to be explored but may be related to the suppression of angiogenesis via VEGF and the inactivation and apoptosis of osteoclasts [37].

Although the periapical lesions have an inflammatory pathology, no traumatic injury was induced in this model, which can explain the lack of osteonecrosis development. As previously described, in patients taking BPs, osteonecrosis is associated with invasive/traumatic dental procedures [19,22], such as tooth extraction, when there is a previous infection (pain, swelling, purulence, fistula) [18] or implants subjected to functional loading with characteristics that trigger osteonecrosis [38].

Some studies have found the development of ONJ in animal models with a periapical lesion; nevertheless, it should be noted that they used higher doses of BPs compared to the quantities that oncologic patients receive, and compared to our study [39]. Therefore, doses equivalent to those in humans should be used in animal models to mimic human disease more accurately, although noticeable differences, namely metabolic, exist between species.

To analyse the development of periapical disease, periapical radiography was used, an essential clinical method to diagnose apical periodontitis and assess the outcome of root canal treatments. Although the two-dimensional representation has limitations and might not strictly reflect biological and clinical features in the bone structures, this is a non-invasive imaging method and is more feasible in clinics [40,41,42,43]. However, the main disadvantage is that mineral loss of approximately 40% is necessary to detect a decrease in radiopacity, so the lesion is only visible in the radiograph when this bone mineral destruction is present [40,41,42].

Thus, a radiographic and histological method was used in this study, combined with nuclear medicine, to allow a more comprehensive evaluation.

Although high-resolution imaging techniques are available nowadays, it would only be possible to image the jaw but not to study bone metabolism. However, through nuclear medicine and using ^99m^Tc-HMDP, it is possible to study bone metabolism with high sensitivity and specificity. Alterations in bone metabolism have repercussions in the jawbone; however, it takes time for these changes to be translated into a bone defect and, consequently, to detect changes in a radiographic assessment.

Nuclear medicine through phosphonate derivatives, which can be localized in bone lesions at a very early stage of the pathologic process, is much more sensitive than conventional X-rays in the early detection of bone lesions and bone necrosis [44,45]. Furthermore, as ^99m^Tc-HMDP is a bone radiotracer that diffuses through capillaries into bone, it becomes strongly bound by chemisorption to the crystals in the bone surface. This distribution reflects both bone inflammation and osteoblastic activity, which can be related to the radiopharmaceutical’s quantitative uptake [44,45,46]. This explains the chemical uptake of BPs in the bone matrix and the impaired effect on osteoclast-mediated bone resorption.

In periapical radiography and computed tomography, a mineral loss of 40–50% is necessary to detect a decrease in radiopacity. In contrast, in nuclear medicine, a change of 5% in bone turnover can be detected [43]. A small amount of bone destruction allows early detection, supporting the important role of nuclear-medicine analysis [47]. This might explain the lack of statistical significance between the groups in the radiology evaluation compared with nuclear medicine, which is more sensitive.

Nevertheless, histological analysis remains the gold standard for accurate diagnosis. In the present work, it corroborates the radiology and nuclear medicine results, presenting wider periapical lesions in the control group compared to the zoledronate group.

The results of this study are in accordance with previous studies performed by Wayama et al. [48] and Rao et al. [49], which induced apical periodontitis in hypoestrogenic rats and in ovariectomized rats (respectively) treated with zoledronic acid, resulting in a decrease in periapical lesion progression [48,49]. Similarly, França et al. [50] showed that rats treated with zoledronic acid had smaller periapical lesions when compared to the control group [50].

Although tooth extraction alone can induce ONJ, a pre-existing periapical lesion increases the probability of MRONJ development [18]. Song et al. [51] and Kang et al. [39] also demonstrated that, in animal models with zoledronate intake, the periapical lesion can be controlled, and they emphasized the importance of that control in the MRONJ development in the case of tooth extraction [39,51].

In the present study, an increase in ^99m^Tc-HMDP radionuclide uptake was seen in periapical lesions. This suggests that zoledronate is present in the alveolar bone infection and supports the effect of ZOL in limiting the area of periapical disease. According to the present findings, the periapical lesions in animals treated with zoledronic acid were significantly smaller when compared to the animals in the control group. These results support the decision that endodontic treatment should be performed instead of extraction to prevent MRONJ, which can be associated with surgical procedures in patients taking BPs, mainly if previous signs of infection are present [18,52]. It should be reinforced that in case of a predictable tooth extraction with a periapical lesion, prior endodontic treatment should be made to decrease the probability of bone histological necrosis. Endodontic treatment will stop the flow of bacteria and their products throughout the apical foramen, healing the periapical lesion [30,52].

Osteonecrosis of the jaw associated with the intake of bisphosphonates is a condition that leads to high morbidity, difficulty eating, pain, swelling and the repeated intake of antibiotics [18,19,20]. Due to the increase in the percentage of medicated patients for oncologic disease or osteoporosis, dentists and other oral health professionals must be aware of which dental procedures represent a risk for the development of osteonecrosis of the jaw. 

There is no consensus that endodontic treatment is safe for patients taking bisphosphonates. Some studies state that endodontic treatment could be a risk factor and that if it is performed, it should not have permeabilization or instrumentation beyond the apex [19]. However, to allow an adequate eradication of microorganisms, permeabilization is essential to give access to the foramen surface and the periapical area and to enable the action of chemical solutions and intracanal medications in the whole length of the root canal. As previously stated, endodontic and periapical lesions present various colonizing microorganism species, including Gram-negative and Gram-positive anaerobes, both strict and facultative, such as *Actinomyces* sp., *Fusobacterium nucleatum* sp., *Prevotella* sp., *Porphyromona gingivalis*, *Streptococcus* sp., and *Propionibacterium* sp. [4,5,6,7]. Some of these species present challenges for eradication, and apex permeabilization can be fundamental to allow complete elimination [1,2,4]. Otherwise, residual microorganisms can persist and lead to future treatment failure. 

To improve the experimental design, in future studies, samples could be collected from the periapical lesions to make a histological evaluation of the alveolar bone and to study the microorganisms present. Further histological evaluation could facilitate understanding cellular interactions and the dynamic between defensive and destructive mechanisms. As future perspectives, determining the microorganisms present could also facilitate understanding the pathobiological basis of cicatrisation in the presence of BP intake. Also, using micro CT could allow more information to be obtained on the bone micro-architecture and early structural changes. Additionally, longer treatment periods with zoledronic acid should be evaluated to assess long-term therapy results in inflammatory periapical lesions and the adjacent alveolar bone.

## 5. Conclusions

In the experimental model used, the presence of microorganisms and periapical periodontitis does not represent a risk factor for developing medication-related osteonecrosis of the jaw. The null hypothesis of this study was accepted as the periapical lesion did not contribute to the establishment of MRONJ. On the contrary, zoledronate therapy inhibits bone resorption in periapical lesions.

## Data Availability

Data are contained within the article.
